# Pre-emptive *CYP2C19* genotyping and voriconazole exposure in pediatric hematology patients at risk of aspergillosis: an exploratory randomized pilot study with a cost-consequence analysis

**DOI:** 10.3389/fphar.2026.1830548

**Published:** 2026-07-21

**Authors:** Jaime Monserrat-Villatoro, David Bueno, Miriam Estébanez, Yasmina Mozo, Rocío Rosas-Alonso, Cristina Antón, María José Otero, Pilar Guerra-García, Xando Díaz-Villamarín, Pedro Arias, Antonio Pérez-Martínez, Francisco Abad-Santos, Antonio J. Carcas, Irene García-García, Alberto M. Borobia

**Affiliations:** 1 Clinical Pharmacology Department, La Paz University Hospital, IdiPAZ, Madrid, Spain; 2 Pediatric Oncology and Haematology Department, La Paz University Hospital, Madrid, Spain; 3 CBRN and Infectious Diseases Department, Hospital Central de la Defensa “Gómez Ulla”, Madrid, Spain; 4 Department of Medicine and Medical Specialties, University of Alcalá, Alcalá de Henares, Spain; 5 Genetic Department, La Paz University Hospital, IdiPAZ, Madrid, Spain; 6 Health Technology Assessment Department, Facultad de Medicina, Universidad Francisco de Vitoria, Pozuelo deAlarcón, Spain; 7 Haematology Department, Hospital Central de la Defensa Gómez Ulla, Universidad de Alcalá, Madrid, Spain; 8 Pharmacology Department, School of Medicine, Universidad Autónoma de Madrid, Madrid, Spain; 9 Clinical Pharmacology Department, Hospital Universitario de La Princesa, Instituto de Investigación Sanitaria La Princesa (IIS-Princesa), Madrid, Spain

**Keywords:** aspergillosis, CYP2C19, pediatric hematology, pre-emptive genotyping, therapeutic drug monitoring, voriconazole

## Abstract

**Introduction:**

Voriconazole is a first-line therapy for invasive aspergillosis, but its use is complicated by pronounced pharmacokinetic variability, largely driven by *CYP2C19* polymorphisms. We aimed to evaluate whether preemptive *CYP2C19* genotyping improves early target attainment and clinical management in pediatric hematology patients compared with standard practice.

**Methods:**

We conducted a pragmatic, randomized, single-blind trial (EudraCT: 2019-000376-41; ClinicalTrials.gov: NCT04238884). Pediatric hematology patients at high risk of aspergillosis were randomized (1:1) to receive either standard weight-based dosing (Control) or preemptive CYP2C19 (*1, *2, *3, *17 alleles) genotype-guided dosing (Experimental). The primary endpoint was the proportion of patients achieving a therapeutic voriconazole trough concentration (Cmin: 1.0–5.0 mg/L) at Day 5 (±1). The analysis was conducted using a modified intention-to-treat approach.

**Results:**

Thirty-two patients (16 per arm) were evaluated. The experimental arm had a higher proportion of patients achieving the therapeutic range compared to the control arm, but not statistically significant (37.5% vs. 12.5%, p = 0.200). The proportion of patients with subtherapeutic exposure (<1.0 mg/L) was lower in the genotype-guided arm than in the standard-of-care arm (31.3% vs. 68.8%, p = 0.034). Supratherapeutic levels were comparable between groups (25% vs. 18.8%, p > 0.900). An exploratory cost-consequence analysis was associated with a mean difference in direct medical costs of €5,819 per patient in favour of the genotype-guided arm, although this difference was not statistically significant (p = 0.300).

**Conclusion:**

Preemptive *CYP2C19* genotyping was associated with a lower proportion of early subtherapeutic voriconazole exposure compared with standard care, with no signal of increased toxicity.

**Clinical Trial Registration:**

https://clinicaltrials.gov/study/NCT04238884; https://www.clinicaltrialsregister.eu/ctr-search/trial/2019-000376-41/ES, identifier EudraCT: 2019-000376-41; NCT04238884.

## 1 Introduction

Invasive aspergillosis, caused by the infection of Aspergillus ssp, is one of the most important causes of morbidity and mortality in patients with hematological diseases. Patients at increased risk of aspergillosis are those with prolonged neutropenia induced by chemotherapy drugs, acute leukemia and those receiving allogenic hematopoietic stem cells transplantation. Incidence of probable or proven invasive aspergillosis in these patients can reach 6%–11% (Koehler et al., 2017; Lestrade et al., 2018), with 27% mortality rates in acute leukemia patients and up to 40% in patients with malignant hematological disease (Pagano et al., 2006). While great advances in the treatment of aspergillosis have been made, the disease remains associated with high morbidity and mortality (Boyer et al., 2023).

Voriconazole is a broad-spectrum triazole used as first-line therapy for invasive aspergillosis and as prophylaxis in high-risk immunocompromised patients, including those undergoing hematopoietic stem cell transplantation or intensive chemotherapy. It exerts its antifungal activity mainly by inhibiting the fungal 14α-sterol demethylase (CYP51), a key enzyme in ergosterol biosynthesis. Voriconazole displays a well-recognized exposure–response relationship, with trough concentrations associated with both clinical efficacy and toxicity. Early attainment of target exposure appears particularly relevant in invasive disease, whereas available evidence suggests that lower trough concentrations may be sufficient for prophylaxis than for treatment of established infection (Dolton et al., 2012; Park et al., 2012; Pascual et al., 2012; Troke et al., 2011; Andes et al., 2009). Current recommendations commonly define a therapeutic trough concentration range of 1–5 mg/L to maximize effectiveness while minimizing adverse events (Lewis et al., 2015). However, achieving and maintaining target exposure early in therapy remains challenging in routine practice due to pronounced pharmacokinetic variability.

Voriconazole is characterized by nonlinear pharmacokinetics and high interindividual variability (Hope, 2012), mainly caused by genetic factors (Moriyama et al., 2017). Approximately 75% of voriconazole is metabolized in the liver, mainly by CYP2C19 and to a lesser extent by CYP3A4 and CYP2C9; the remaining 25% is metabolized by the Flavin-containing monooxygenase (FMO) protein family. Approximately 50% of the interindividual variability in voriconazole metabolism is caused by single nucleotide polymorphisms (SNPs) in *CYP2C19* (Weiss et al., 2009). 5%–17% of the patients treated with voriconazole are ultra-rapid metabolizers (UM; CYP2C19*17/*17 genotype) and between 25% and 33% are rapid metabolizers (RM; CYP2C19*1/*17), which, in both cases, results in risk of suboptimal concentrations of the drug in patients’ serum (Hicks et al., 2014; Dapía et al., 2019). 2%–5% of Caucasian patients are poor metabolizers and show 4-fold higher levels of voriconazole than normal metabolizers (NM) (Dean et al., 2012).

Current guidance and consensus documents support therapeutic drug monitoring (TDM) of voriconazole and acknowledge *CYP2C19* genotype as a clinically relevant determinant of exposure (Chau et al., 2021; Douglas et al., 2021). However, recommendations on how to operationalize pharmacogenetic information vary across guideline bodies, ranging from considering alternative agents for extreme metabolizer phenotypes to phenotype-guided dose modifications, typically in conjunction with TDM (Moriyama et al., 2017; Dutch Pharmacogenetics Work ing Group, 2018). Model-informed analyses further suggest that clinically meaningful, phenotype-dependent dose differences may be required to achieve target exposure, underscoring the need to prospectively evaluate implementable strategies in real-world settings (Zubiaur et al., 2021).

In practice, the pronounced interindividual variability and non-linear pharmacokinetics of voriconazole can delay attainment of target concentrations during the initial days of therapy, when early exposure may be critical for both effectiveness and tolerability (García et al., 2020). A pragmatic approach to address this gap is to make actionable pharmacogenetic information available before voriconazole initiation in populations with predictable risk of exposure extremes.

We therefore hypothesize that pre-emptive *CYP2C19* genotyping in hematological patients at risk of aspergillosis can improve early target attainment and clinical management compared with standard practice. To test this hypothesis, we conducted a randomized clinical trial evaluating the effectiveness and efficiency of a pre-emptive genotyping strategy to guide the initial voriconazole dosing regimen in patients receiving voriconazole for prophylaxis or treatment of invasive aspergillosis.

## 2 Methods

### 2.1 Study design and ethical oversight

We conducted a phase IV, pragmatic, multicenter, randomized, single-blind, active-controlled clinical trial to evaluate a pharmacogenetics-guided dosing strategy for voriconazole. The study protocol was approved by the Clinical Research Ethics Committee of La Paz University Hospital (Internal Code: 5316) and authorized by the Spanish Agency of Medicines and Medical Devices (AEMPS). The trial was conducted in strict compliance with the Declaration of Helsinki and Good Clinical Practice (GCP) guidelines.

The study was registered in the European Union Drug Regulating Authorities Clinical Trials Database (EudraCT: 2019-000376-41) and ClinicalTrials.gov (NCT04238884). The full protocol has been previously published (Monserrat Villatoro et al., 2020).

This trial was designed as a confirmatory efficacy study. It was conducted under a public competitive grant (ISCIII PI18/01322) with a pre-specified execution period. The SARS-CoV-2 pandemic substantially delayed the initiation of recruitment, and the planned sample size of 146 patients could not be reached within the fixed grant period; recruitment was therefore closed when the allocated execution period ended, and not due to safety concerns or futility. Consequently, the analysis presented here is the final analysis of the recruited pediatric population.

### 2.2 Participants

The study population consisted of pediatric patients (aged 2–17 years) recruited from the Department of Pediatric Hemato-Oncology at La Paz University Hospital. Eligible participants were children at high risk of invasive aspergillosis (IA) scheduled to receive voriconazole for either prophylaxis or treatment. Specific inclusion criteria included: diagnosis of acute myeloid leukemia (*de novo* or relapsed), relapsed acute lymphoblastic leukemia, or patients undergoing allogeneic hematopoietic stem cell transplantation (HSCT).

Patients were excluded if they had a history of hypersensitivity to voriconazole or if they were participating in another clinical trial that prohibited concomitant medication. Written informed consent was obtained from parents or legal guardians, and assent was obtained from patients aged 12 years or older, prior to any study procedure.

### 2.3 Randomization and masking

Participants were randomly assigned (1:1 ratio) to either the Control Group (Standard of Care) or the Experimental Group (Genotype-Guided Dosing) using a centralized electronic Case Report Form (eCRF) with a randomization sequence generated by SAS V.9.4, it was subsequently uploaded to REDCap (Research Electronic Data Capture), which served as the centralized electronic Case Report Form (eCRF) to manage the allocation process. Randomization was stratified by center.

Due to the pragmatic nature of the intervention (dose adjustment), treating physicians and clinical pharmacologists were aware of group allocation. However, patients and their families remained blinded to the study arm assignment throughout the treatment period.

### 2.4 Interventions and dosing algorithms

Voriconazole (VFEND®, Pfizer, or bioequivalent generic formulations) was administered intravenously (IV) or orally (PO) based on clinical status. Bioequivalence between oral and IV formulations was assumed to be high (≥96%) in accordance with the summary of product characteristics (SmPC) (European Medicines Agency EMA, 2026).

#### 2.4.1 Control group (standard of care)

Patients assigned to the control group received voriconazole dosing strictly according to total body weight and age, following the standard manufacturer’s Summary of Product Characteristics (SmPC) (European Medicines Agency EMA, 2026) and institutional guidelines (Ashbee et al., 2014). For both intravenous and oral administration, an initial loading dose of 9 mg/kg every 12 h was administered during the first 24 h. This was followed by a maintenance regimen consisting of 8 mg/kg every 12 h for the intravenous route, or 9 mg/kg every 12 h (up to a maximum of 350 mg per dose) for oral administration. In this group, dose adjustments were not performed preemptively, but were managed reactively based on the results of the TDM assessment conducted on Day 5 (±1).

#### 2.4.2 Experimental group (genotype-guided)

In the intervention group, voriconazole dosing was preemptively adjusted according to the patient’s *CYP2C19* phenotype, which was identified during the screening phase. The dosing algorithm was developed by integrating CPIC guidelines with published pediatric pharmacokinetic data. Specifically, Normal Metabolizers (NM; *1/*1) received the standard dosing regimen, identical to that of the control group. For Rapid (RM; *1/*17) and Ultrarapid Metabolizers (UM; *17/*17), a dose intensification of 50%–100% was implemented to mitigate the risk of underexposure, consisting of 15–18 mg/kg every 12 h for both loading and maintenance phases. Conversely, Poor Metabolizers (PM; e.g., *2/*2, *2/*3) underwent a 25% dose reduction (6–7 mg/kg every 12 h) to prevent potential toxicity; in these cases, an optional TDM was permitted at 24–48 h for early safety screening. Following this initial genotype-guided approach, all subsequent dose modifications from Day 5 (±1) onwards were managed reactively based on TDM results.

### 2.5 Genotyping and TDM

Genotyping was performed at the Institute of Medical and Molecular Genetics (INGEMM) using the PharmArray V.2 platform, a custom-designed SNP array. The analysis specifically targeted key *CYP2C19* variants, including the loss-of-function alleles *2 (rs4244285) and *3 (rs4986893), and the gain-of-function allele *17 (rs12248560). The wild-type *1 allele was assigned by default in the absence of these variants. Phenotypic classification was subsequently determined following the standard Clinical Pharmacogenetics Implementation Consortium (CPIC) definitions.

Regarding the pharmacokinetic assessment, TDM was conducted by measuring serum voriconazole trough concentrations (Cmin) from samples collected 5–15 min prior to the morning dose on Day 5 (±1), ensuring steady-state conditions. Quantitative analysis was carried out using a validated chemiluminescent microparticle immunoassay (CMIA) on the Abbott ARCHITECT c4000 system. In alignment with institutional Standard Operative Procedures (SOPs) and evidence from pivotal clinical cohorts, the target therapeutic range was defined as 1.0–5.0 mg/L. This limit reflects the mean maximum threshold associated with efficacy and safety convergence across the literature (Dolton et al., 2012; Park et al., 2012; Pascual et al., 2012; Troke et al., 2011).

### 2.6 Data management and monitoring

Clinical data were collected using a validated electronic Case Report Form (eCRF) designed with MACRO Clinical Trial Management Software. The eCRF was developed to capture all study variables, while ensuring strict pseudonymization; no direct personal identifiers were stored in the study database.

Data management and quality control were performed by the Spanish Clinical Research Network (SCReN) in adherence to the approved Data Management Plan. This included programmed consistency checks, query generation for missing or inconsistent data, and a final audit prior to database lock. Once validated, data were exported to standardized formats for statistical analysis.

### 2.7 Outcomes

The primary endpoint of this study was the proportion of patients achieving therapeutic serum voriconazole concentrations, defined as a Cmin between 1.0 and 5.0 mg/L, at steady state on Day 5 (±1). Secondary pharmacological objectives included the incidence of subtherapeutic (<1.0 mg/L) and supratherapeutic (>5.0 mg/L) levels across both study arms.

Safety was evaluated through the systematic recording and monitoring of adverse events (AEs). Following the study protocol, the intensity of these events was categorized into three grades: mild, where discomfort was observed but did not alter normal daily activity; moderate, characterized by discomfort sufficient to reduce or affect normal daily activity; and severe, defined as the incapacity to perform standard daily activities.

Finally, an economic evaluation was prespecified in the trial protocol. It was originally planned as a Spanish National Health System perspective cost-effectiveness analysis. Given the limited sample size of the analyzable cohort, it is reported here as an exploratory cost-consequence analysis. Real per-patient direct medical costs were calculated from individual healthcare resource use over the 90-day follow-up period of each participant, multiplied by the corresponding unit costs published by the Spanish National Health System and by the Madrid regional Department of Health for the year of the trial. The cost components included hospitalization days (general ward and intensive care, as applicable), outpatient and emergency visits, diagnostic tests, antifungal medications, voriconazole therapeutic drug monitoring, and the pre-emptive *CYP2C19* genotyping procedure itself. For each patient, all these components were summed into a single direct medical cost figure, and the mean and dispersion of these per-patient costs were then compared between the two arms. In a separate descriptive layer, the per-patient costs were also stratified according to the achievement of the primary pharmacological endpoint (therapeutic, subtherapeutic and supratherapeutic categories), in order to explore whether the cost profile differed across these clinical outcomes within each arm. No QALYs or incremental cost-effectiveness ratios were derived, given the underpowered nature of the analysis.

### 2.8 Statistical analysis

The initial sample size was prespecified in the published protocol calculated at 146 patients to detect an absolute increase in the proportion of patients within the therapeutic range on day 5 from 43.3% under standard care to 68% under the genotype-guided strategy (two-sided α = 0.05, 80% power, 62 evaluable patients per arm; inflated to 146 to account for an estimated 85% of consented patients ultimately receiving voriconazole). The 43.3% baseline figure and the 68% expected proportion were derived from the pediatric cohort and the population simulation reported by Hicks et al., which already integrate the expected distribution of *CYP2C19* metabolizer phenotypes (NM, IM, RM, UM, PM) in the underlying population (Hicks et al., 2014). However, due to the early termination of the trial, the final analysis was conducted on the available cohort of 32 patients (N = 16 per arm) and is reported as an exploratory, hypothesis-generating analysis.

Both efficacy and safety data were analyzed using a modified Intention-To-Treat approach (Ashbee et al., 2014). The final analysis population consisted of 32 subjects who completed the required procedures, excluding 4 adults and 13 pediatric patients due to insufficient samples or screening failures. The primary pharmacological endpoint was the achievement of therapeutic voriconazole trough levels (Cmin) at Day 5 (±1). Descriptive statistics were used to summarize baseline demographics and clinical characteristics.

Categorical variables were compared using Pearson’s chi-squared test when the number of subjects in both groups was greater than 5; otherwise, Fisher’s exact test was applied. Continuous variables were assessed with the Mann-Whitney U test (Wilcoxon rank-sum test) given the small sample size and non-normal distribution of the data. Risk estimates for effectiveness were expressed as Odds Ratios (OR) and Relative Risk (RR) with 95% Confidence Intervals (CI). All statistical procedures were performed using R software (version 4.3.1), with a two-sided p-value <0.05 established as the threshold for statistical significance.

## 3 Results

### 3.1 Study population

A total of 49 candidates with high-risk hematological conditions were screened between January 2020 and July 2023 across the participating centers. As specified in the original protocol^22^, the trial was initially designed to enrol both adult and paediatric patients at risk of invasive aspergillosis; for the paediatric population, the protocol did not predefine a strict lower age limit, leaving the inclusion of younger children to the clinical judgement of the investigators in each participating site. The recruitment phase was significantly impacted by the operational constraints of the SARS-CoV-2 pandemic, which necessitated strategic adjustments to the final study population. Following rigorous screening, 17 subjects were excluded. Specifically, four adult patients recruited at Hospital Central de la Defensa “Gómez Ulla” were not retained in the final analysis because the pandemic-related disruptions, together with the progressive introduction during the recruitment period of newer antifungal agents (most notably isavuconazole and posaconazole) into routine adult haematology practice, which displaced voriconazole as a first-line option in this population, prevented the accrual of a statistically powered adult sample; we therefore restricted the present analysis to the paediatric cohort, in which voriconazole remained the first-line antifungal of choice throughout the recruitment period, in order to preserve pharmacokinetic homogeneity. Additionally, 12 pediatric candidates were excluded due to not requiring voriconazole therapy following baseline genotyping, and 1 was an inclusion failure, excluded prior to randomization.

**FIGURE 1:**
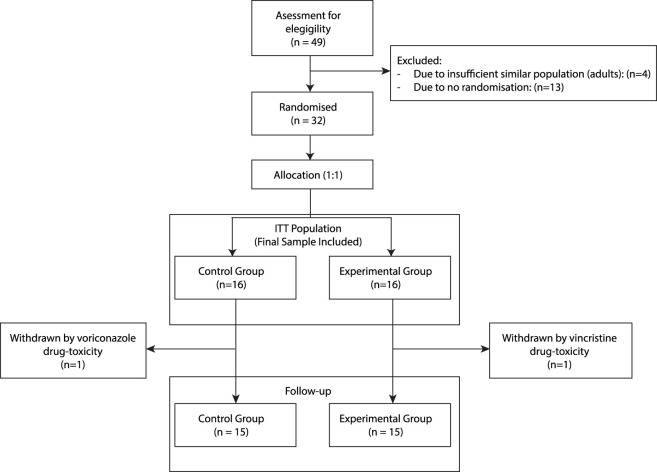
CONSORT flow diagram.

Consequently, 32 pediatric patients were randomized in a 1:1 ratio into the control (n = 16) and experimental (n = 16) groups using a centralized electronic system stratified by center. All randomized participants were retained for the modified Intention-to-Treat (mITT) analysis (Gupta, 2011). Two discontinuations occurred during the active treatment phase: one control patient (ID 28) was withdrawn due to suspected hepatotoxicity, and one experimental patient (ID 15) discontinued the study drug to initiate chemotherapy with vincristine, in accordance with protocol safety measures to avoid severe drug-drug interactions (See Figure 1).

### 3.2 Baseline characteristics

Baseline characteristics for the 32 evaluable pediatric patients are summarized in Table 1. The randomization process yielded a study population that was well-balanced regarding demographic variables. The median age of the overall cohort was 8.9 years (IQR: 5.4–12.8), with no statistically significant differences observed between the control (8.4 years) and experimental (9.8 years) arms (p = 0.12). Similarly, sex distribution was symmetrical (p = 0.7), comprising 53.1% males and 46.9% females across the total cohort. The study population reflected the ethnic diversity of the participating centers, with a predominance of Caucasian (56.2%) and Latin American (34.4%) subjects, alongside smaller representations from other regions; this distribution was comparable between arms (p = 0.3), minimizing potential bias from population-specific genetic variability outside the tested *CYP2C19* alleles.

**TABLE 1:** Demographic and anthropometric data.

Variable	Total (N = 32)^a^	Control (N = 16)^a^	Experimental (N = 16)^a^	p-value
Age (years) Median (IQR)	8.9 (5.4, 12.8)	8.4 (1.6, 11.0)	9.8 (6.8, 14.1)	0.12^a^
Sex N (%)	​	​	​	0.7^b^
Male	17 (53.1%)	9 (56.3%)	8 (50%)	---
Female	15 (46.9%)	7 (43.8%)	8 (50%)	---
Ethnic origin N (%)	​	​	​	0.3^c^
Caucasian	18 (56.2%)	11 (68.8%)	7 (43.8%)	---
Latin American	11 (34.4%)	4 (25.0%)	7 (43.8%)	---
Mid orient	1 (3.1%)	0 (0%)	1 (6.3%)	---
North Africa	1 (3.1%)	0 (0%)	1 (6.3%)	---
East Asia	1 (3.1%)	1 (6.3%)	0 (0%)	---
Weight (kg) Median (IQR)	31 (20, 42)	23 (16, 31)	37 (24, 48)	0.02^d^
Height (cm) Median (IQR)	128 (111, 148)	124 (80, 145)	134 (119, 151)	0.3^d^

^a^ Wilcoxon rank sum exact test.

^b^ Pearson’s Chi-squared test.

^c^ Fisher’s exact test.

^d^ Wilcoxon rank sum test.

However, a statistically significant imbalance was identified in anthropometric measurements (p = 0.02). The control group presented a significantly lower median body weight (23 kg; IQR: 16–31) compared to the experimental group (37 kg; IQR: 24–48). This baseline discrepancy is relevant for the interpretation of pharmacokinetic outcomes, as lower body weight in pediatric populations is frequently associated with higher metabolic rates per kilogram due to allometric scaling principles and liver-to-body-mass ratios (Friberg et al., 2012; Walsh et al., 2004). Given the small sample size, this imbalance can plausibly be attributed to chance, and any pharmacokinetic implication should be interpreted with caution.

Beyond these demographic and anthropometric parameters, the study population exhibited a high degree of clinical complexity consistent with tertiary pediatric hematology-oncology care. The primary indications for antifungal management were severe hematological drivers, predominantly Acute Lymphoblastic Leukemia (ALL), Acute Myeloid Leukemia (AML), and bone marrow failure syndromes requiring intensive immunosuppression.

Regarding comorbidities with potential pharmacokinetic impact, a notable asymmetry was observed in hepatic status. Diagnoses potentially affecting drug metabolism, specifically liver fibrosis and hepatoblastoma, were exclusively present in the Control arm. As with the body-weight imbalance described above, this asymmetry should be interpreted with caution given the small sample size, since differences of this magnitude can occur by chance in cohorts of this size. We have therefore avoided drawing mechanistic inferences about its potential pharmacokinetic direction or its effect on the primary endpoint. Other comorbidities, including renal insufficiency and combined immunodeficiencies, were sporadically distributed and showed no statistically significant patterns that would confound the interpretation of the primary endpoint.

### 3.3 Pharmacogenetic profile

Complementing the clinical assessment, *CYP2C19* genotyping was successfully performed for all randomized participants using the PharmArray v.2 platform. The analysis interrogated key alleles determining metabolic function, specifically loss-of-function (*2, *3) and gain-of-function (*17) variants. Stratification according to Clinical Pharmacogenetics Implementation Consortium (CPIC) guidelines revealed a substantial prevalence of clinically relevant pharmacogenetic variants across the study population (Moriyama et al., 2017).

In the Control group (n = 16), the majority of patients were classified as Normal Metabolizers (n = 10, 62.5%). The remaining participants exhibited altered metabolic profiles: three patients (18.8%) were identified as Intermediate Metabolizers, characterized by reduced enzymatic activity. Simultaneously, an equal subset (n = 3, 18.8%) carried gain-of-function alleles resulting in Rapid or Ultrarapid phenotypes. Consequently, nearly 40% of the standard-of-care arm entered the study with genetically determined deviations in clearance—specifically, nearly one in five patients was predisposed to accelerated metabolism and subtherapeutic serum concentrations, while another one patient faced a potential theoretical risk of accumulation.

Conversely, the Experimental group (n = 16) presented a distinct metabolic risk profile. While half of the patients were Normal Metabolizers (n = 8, 50%), the remaining population exhibited a broad spectrum of altered metabolism. The group included an equal proportion of Intermediate and Rapid Metabolizers (n = 3, 18.8% each). Most critically, this arm exclusively contained the cohort’s Poor Metabolizers (n = 2, 12.5%). This specific distribution was pivotal for the study’s safety objectives, as the patients at the highest risk for concentration-dependent toxicity were allocated to the genotype-guided arm, triggering the protocol-mandated dose reduction or alternative therapy.

The observed genotype distribution complied with Hardy-Weinberg equilibrium (p > 0.05). The minor allele frequencies (MAF) for the loss-of-function CYP2C19*2 (rs4244285) and the gain-of-function CYP2C19*17 (rs12248560) variants were 15.6% and 12.5%, respectively. These values are consistent with reference data for Ad-mixed American and European populations in the gnomAD v4 database (Karczewski et al., 2020), which reports MAFs ranging from 13%–15% for 2% and 13%–20% for 17. The absence of the CYP2C19*3 allele (rs58973490) in our cohort also aligns with its negligible prevalence (<0.5%) in these demographic groups, confirming the external validity of the study’s pharmacogenetic profile.

### 3.4 Efficacy results

Against this background of clinical and metabolic complexity, the primary objective of the study was to evaluate the proportion of patients achieving a steady-state serum voriconazole trough concentration (Cmin) within the therapeutic window of 1.0–5.0 mg/L on Day 5. This conservative range aligns with the strict safety limits proposed by the British Society for Medical Mycology (BSMM) (Ashbee et al., 2014) and falls within the broader recommendations of the ECIL-6 guidelines (Tissot et al., 2017).

The modified Intention-to-Treat (mITT) (Ashbee et al., 2014) analysis demonstrated a positive trend in target attainment, with the Experimental group achieving a success rate of 37.5% (6/16) compared to only 12.5% (2/16) in the Control group. Although this difference did not reach statistical significance (p = 0.200) due to limited statistical power (1−β = 0.597), a deeper interrogation of the data reveals a statistically significant benefit in risk mitigation.

The pivotal finding emerged from analyzing the direction of therapeutic failures. The standard-of-care arm exhibited a profound tendency toward underdosing, with 68.8% (11/16) of Control patients falling into the subtherapeutic range (<1.0 mg/L), exposing them to a high risk of invasive fungal breakthrough. In contrast, the prevalence of subtherapeutic exposure was lower in the genotype-guided arm (31.3%; 5/16; p = 0.034, Fisher’s exact test). The *post hoc* power was approximately 0.65 for the dichotomized comparison of subtherapeutic exposure. Given the small cohort, this signal should be interpreted as exploratory and hypothesis-generating; nevertheless, it is consistent with the pharmacological rationale of pre-emptive *CYP2C19* genotyping, which is to provide actionable information before voriconazole initiation in order to reduce the proportion of patients exposed to early underdosing (Table 2).

**TABLE 2:** Voriconazole trough concentrations at TDM sampling on Day 5.

Outcome category	Total n (%) (N = 32)	Control n (%) (N = 16)	Experimental n (%) (N = 16)	p-value
Therapeutic (1.0–5.0 mg/L)	8 (25%)	2 (12.5%)	6 (37.5%)	0.200^a^
Subtherapeutic (<1.0 mg/L)	16 (50%)	11 (68.8%)	5 (31.3%)	0.034^b^
Supraterapeutic (>5.0 mg/L)	7 (21.9%)	3 (18.8%)	4 (25%)	>0.900^a^
Unknown/Missing	1 (3.1%)	0 (0%)	1 (6.3%)	>0.900^a^

^a^ Fisher’s exact test.

^b^ Pearson’s Chi-squared test.

We acknowledge the conceptual limitations of *post hoc* power analysis and report these values descriptively.

### 3.5 Safety results

A primary safety concern regarding genotype-guided dosing—specifically the recommendation to escalate doses by 50%–100% in Ultrarapid Metabolizers—is the potential for unintended toxicity. The trial results alleviate this concern, demonstrating that aggressive up-titration in identified rapid metabolizers did not result in a disproportionate rate of toxic accumulation. The incidence of supratherapeutic serum levels (>5.0 mg/L) was statistically comparable between the two arms (25% in the Experimental group vs. 18.8% in the Control group; p > 0.90; Table 2).

The safety utility of the intervention was further validated by the management of the high-risk Poor Metabolizers (PM, CYP2C19 *2/*2) identified in the Experimental group. Under the study protocol, these patients were flagged pre-emptively, triggering a clinical decision to reduce the voriconazole starting dose by at least 25%, followed by early therapeutic drug monitoring (TDM) within 24–72 h (Hicks et al., 2014). While current guidelines also consider the option of switching to an alternative antifungal agent not dependent on CYP2C19 metabolism for these extreme phenotypes (Moriyama et al., 2017), patients who were entirely switched to an alternative therapy did not meet the requirement for randomization (which strictly required voriconazole administration) and were subsequently excluded from modified Intention-to-Treat analysis. Consequently, the analyzed PM patients, managed with preemptive dose reductions, did not experience severe toxicity.

The pharmacokinetic safety profile translated into a favorable clinical tolerability profile over the 90-day follow-up. The overall burden of adverse events (AEs) was comparable between arms, with 25% of Experimental patients (4/16) and 31% of Control patients (5/16) reporting at least one AE (p > 0.900) (Table 3). While the sample size limits definitive conclusions, these data suggest that the genotype-guided strategy did not generate a discernible signal of increased iatrogenic morbidity in this cohort.

**TABLE 3:** Number and percentage of adverse events, and patients reporting adverse events.

Event	Total n (%)	Control n (%)	Experimental n (%)	p-value^a^
Adverse events reported				
AE	16 (100.0%)	9 (100.0%)	7 (100.0%)	---
SAE	1 (6.3%)	0 (0.0%)	1 (14.0%)	0.400
ADR	1 (6.3%)	0 (0.0%)	1 (14.0%)	0.400
Patients reporting adverse events				
AE	9 (28.1%)	5 (31.0%)	4 (25.0%)	>0.900
SAE	1 (3.1%)	0 (0.0%)	1 (6.3%)	>0.900
ADR	1 (3.1%)	0 (0.0%)	1 (6.3%)	>0.900

AE: Adverse Event; SAE: Serious Adverse Ev^ent; ADR: Adverse Drug Reaction.^

^a^ Fisher’s exact test.

#### 3.5.1 Specific event breakdown

To ensure full transparency regarding the clinical course of the participants, all recorded adverse events were categorized according to MedDRA terms. In the Control group (9 total events), the safety notifications consisted of febrile disorders (n = 4), one episode of sepsis, one bleeding event, one vomiting episode, one dehydration event, and one uncategorized event. In the Experimental group (7 total events), notifications included infections and infestations (n = 3), one febrile disorder, and one case of hemorrhagic cystitis. These patterns are consistent with the underlying high-risk hematological conditions of the study population rather than voriconazole toxicity.

Serious Adverse Events (SAEs) and Reactions Regarding severity, only one Serious Adverse Event (SAE) was reported in the entire cohort: an episode of anaphylactic shock in the Experimental group (1/16, 6.3%). The clinical causality assessment determined this event was unrelated to the study drug. Additionally, one mild-to-moderate Adverse Drug Reaction (ADR) consisting of liver function test disturbance was confirmed in the Experimental group (1/16, 6.3%), which did not require permanent discontinuation of therapy. No SAEs or confirmed ADRs were recorded in the Control group (p = 0.400 for both comparisons).

#### 3.5.2 Discontinuations

The analysis of discontinuations highlights a critical divergence in safety management. In the Control group, one patient (6.3%) required permanent withdrawal of voriconazole due to suspected drug-induced toxicity, confirmed by a supratherapeutic concentration of 7.1 mg/L. In contrast, no discontinuations due to toxicity occurred in the Experimental group; the single withdrawal in this arm was a “proactive” protocol-mandated measure to avoid a severe drug-drug interaction with vincristine, thereby preventing toxicity before it could occur.

### 3.6 Pharmacoeconomic and efficiency analysis

To evaluate the efficiency of the intervention, a cost-consequence analysis was performed from the perspective of the Madrid Regional Health Service, accounting for direct medical costs (hospitalizations, medical visits, diagnostic tests, genotyping) based on public prices published in the Official Gazette of the Community of Madrid (Table 4) (Comunidad de Madrid, 2023).

**TABLE 4:** Description of costs incurred during the follow-up period.

Type of assistance	Description	Unit price (EUR)	Control n	Experimental n
Consultation	Follow-up in-person consultations	79.0	11	9
Consultation	First in-person consultations	132.0	3	2
Day hospital	Consultation + pharmacological treatment session	830.0	37	18
Inpatient	Major hematological/Immunological diagnoses except sickle cell crisis and coagulation severity 1	5583.0	0	1
Inpatient	Acute leukemia severity 1	6625.0	9	12
Inpatient	Acute leukemia severity 2	12722.0	5	2
Inpatient	Acute leukemia severity 3	27017.0	3	0
Inpatient	Lymphatic neoplasms and others and neoplasms of uncertain behavior severity 1	3465.0	1	0
Inpatient	Lymphatic neoplasms and others and neoplasms of uncertain behavior severity 3	6053.0	1	0
Diagnostic test	Pharmacogenetic testing (*CYP2C19* genotyping)	120	0*	16
Diagnostic test	Neck ultrasound (thyroid, parotid, submandibular)	48.72	0	1
Diagnostic test	Chest ultrasound	48.72	2	2
Diagnostic test	MRI without contrast	130.0	1	0
Diagnostic test	Abdomino-pelvic CT without contrast	64.40	1	0
Diagnostic test	Positron emission tomography (PET-CT) with acetate-C11	113.0	2	0
Readmission	Acute leukemia severity 2	12722.0	5	2
Readmission	Acute leukemia severity 3	27017.0	3	1
Emergency	Pediatric emergency	137.0	3	0

* Although *CYP2C19* genotyping was performed in both study arms to ensure clinical safety, the cost of the pharmacogenetic assay was exclusively imputed to the experimental group for the purpose of the economic evaluation.

The analysis was associated with a numerically lower direct medical cost in the genotype-guided arm, although the difference did not reach statistical significance. The mean total direct cost per patient was €9,600 (SD: €10,986) in the Experimental group compared to €15,419 (SD: €16,389) in the Control group, representing a mean difference of €5,819 per patient in favour of the experimental arm (p = 0.300, Wilcoxon-Mann-Whitney; Cohen’s d = 0.42). Given the high inter-individual dispersion of hematological costs, a non-parametric bootstrap analysis (10,000 replicates) estimated a mean difference of €5,746.89 (95% CI: −€3,020 to +€15,886), whose confidence interval crosses zero. These exploratory results should therefore be interpreted as suggesting a potential reduction in direct medical costs that requires confirmation in adequately powered studies.

In addition, patients in the Experimental arm required fewer consultations (3.1 vs. 5.8), day hospital sessions (1.13 vs. 2.31), and emergency visits (0.00 vs. 0.19) compared to the Control arm.

Critically, the drivers of this economic efficiency become evident when stratified by clinical outcome (Table 5). In the Control group, therapeutic failure imposed a disproportionate and highly variable economic burden. Patients with subtherapeutic levels incurred a median cost of €11,902 (IQR: €6,957 – €18,744), while those with supratherapeutic levels reached a median of €26,573 (IQR: €16,599 – €36,548), reflecting the intense resource consumption required to manage complications such as toxicity-induced withdrawals.

**TABLE 5:** Cost by group.

Outcome category	Total N = 32	Control n = 16	Experimental n = 16	p-value
Cost^a^	-	15,419 (16,389)	9,600 (10,986)	0.300^b^
Cost depending on efficacy^c^				
Within therapeutic range	9,115.00 [6,625.00, 19,347.00]	9,290.00 [9,202.50, 9,377.50]	8,903.00 [6,625.00, 19,347.00]	​
Subtherapeutic	7,955.00 [6,625.00, 16,936.00]	11,902.50 [6957.50, 18,744.35]	6625.00 [6,625.00, 6,757.00]	​
Supratherapeutic	6,886.00 [6,704.00, 11,605.00]	26,573.50 [16599.25, 36,547.75]	6886.36 [6,799.79, 8,107.00]	​

^a^ Mean (SD).

^b^ Wilcoxon-Mann-Whitney Test (Exact).

^c^ Median [Q1, Q3].

In the Experimental group, the median cost for subtherapeutic patients was €6,625 (IQR: €6,625 – €6,757) and €6,886 (IQR: €6,799 – €8,107) for supratherapeutic patients, with notably narrower interquartile ranges than in the Control arm. These observations are descriptive and were not pre-specified for inferential testing; given the limited sample size and the wide cost dispersion described above, any difference between arms in the management of therapeutic deviations should be interpreted with caution and considered hypothesis-generating.

## 4 Discussion

The management of invasive fungal diseases (IFD) in pediatric hematology remains a clinically challenging scenario, in which voriconazole exposure is influenced by considerable inter-individual pharmacokinetic variability. Voriconazole is supported as first-line therapy by ECIL-6 and BSMM guidelines, with TDM commonly used to refine dosing during treatment. The VORIGENIPHARM trial was designed to explore whether complementing this approach with a pre-emptive genotype-guided strategy at the moment of treatment initiation might be feasible and informative. In this exploratory analysis, integrating *CYP2C19* genotyping into the initial dosing algorithm was associated with a lower proportion of patients with early subtherapeutic exposure compared with standard care. The study did not reach statistical significance for its primary endpoint, and the observed direction in costs was also not statistically significant. These findings should be regarded as exploratory and hypothesis-generating, and they require confirmation in adequately powered multicentre studies.

It is well established that pediatric patients are not simply “small adults” from a pharmacokinetic perspective. They display distinct developmental pharmacokinetics, with maturational changes in hepatic ontogeny and in cytochrome P450 activity that vary across age strata and are not fully captured by linear weight-based dosing or by allometric scaling alone. In the Control group of our cohort, nearly 70% of patients failed to reach the therapeutic threshold (>1.0 mg/L) by Day 5, which is consistent with the inadequacy of standard weight-based regimens previously described in pediatric voriconazole pharmacokinetic studies. Our trial was not designed or powered to disentangle the relative contribution of age-related developmental pharmacokinetics, body weight, and *CYP2C19* genotype to this high rate of underdosing, and the limited sample size precludes a meaningful stratified analysis by paediatric age subgroups. These observations should therefore be interpreted with caution.

The primary objective of the study was to evaluate target attainment within the strict range of 1.0–5.0 mg/L. While the genotype-guided arm achieved nearly triple the success rate of the control arm (37.5% vs. 12.5%), this difference did not reach statistical significance for the specific interval (p = 0.200). This lack of statistical power must be interpreted in the context of the study’s limited sample size, which was severely curtailed by the unprecedented systemic disruption of clinical research during the COVID-19 pandemic. Rather than a mere temporary cessation of recruitment, the pandemic triggered a global and profound realignment of medical research and healthcare priorities. The vast majority of clinical and financial resources, as well as research personnel, were abruptly diverted away from practically all non-COVID-19 fields—including oncology, hematology, and their associated clinical trials—to focus on the management of SARS-CoV-2. Consequently, non-COVID-19 trials worldwide faced massive suspensions and insurmountable recruitment barriers that precluded reaching initially planned sample sizes (Comunidad de Madrid, 2023; Sohrabi et al., 2021). However, viewing efficacy solely through this binary lens obscures the true clinical value of the intervention.

The most relevant exploratory finding of this analysis relates to risk mitigation. The genotype-guided strategy was associated with a lower prevalence of subtherapeutic levels compared with standard care (31.3% vs. 68.8%, p = 0.034). Although this analysis is exploratory and the trial was underpowered, this signal is consistent with the pharmacological rationale of the intervention, in which actionable pharmacogenetic information is made available before the start of treatment to mitigate the risk of early underexposure in a population in which reaching steady-state exposure quickly may be clinically relevant. These findings should be interpreted as hypothesis-generating and require confirmation in adequately powered studies.

The allele frequencies observed (CYP2C19*2 at 15.6% and *17 at 12.5%) are consistent with gnomAD reference populations for admixed European/Latino demographics, supporting the external validity of the cohort’s pharmacogenetic profile.

Safety was evaluated as a secondary objective, particularly to address clinical concerns that genotype-guided dosing alterations in Ultrarapid or Rapid Metabolizers might inadvertently lead to toxicity. While patients in the experimental arm did not experience a significantly disproportionate rate of supratherapeutic levels compared to the control group (25% vs. 18.8%, p > 0.90), this lack of statistical difference must be interpreted with caution. Due to the limited sample size and the resulting underpowered nature of the study, this equivalence reflects a lack of statistical sensitivity rather than definitive proof of safety. Nevertheless, the trial provided valuable qualitative insights into the critical management of Poor Metabolizers (PM). In the experimental arm, two PM patients were identified *a priori*. Had these subjects been assigned to the Control arm (blind to genotype), they would have received standard weight-based doses known to generate exposure levels up to 4-fold higher than normal in PM phenotypes, which would have placed them at an increased risk of toxicity (Dean et al., 2012). By contrast, in the standard-of-care arm, toxicity management was reactive. For instance, in the control group, toxicity was only addressed after the drug had accumulated to dangerous supratherapeutic levels (e.g., 7.1 mg/L), which ultimately necessitated treatment withdrawal. Given the limited sample size, these observations are illustrative rather than confirmatory, and larger cohorts are needed to evaluate whether pre-emptive genotyping might support a more anticipatory approach to safety management (Moriyama et al., 2017).

A further aspect of this strategy relates to its potential economic implications. As explicitly stated above, the trial was not powered to detect differences in economic outcomes, so all the figures discussed in this section should be interpreted with caution. The exploratory cost-consequence analysis, prespecified in the trial protocol, was associated with a numerically lower direct medical cost in the experimental arm. The available evidence on the economic profile of pharmacogenetic-guided dosing is mixed, and our findings might suggest that *CYP2C19* genotyping might offset these initial costs, pointing towards a potentially favorable economic profile rather than definitively proving it to be a “dominant” intervention (Koufaki et al., 2023). The analysis estimated a mean difference of approximately €5,700 per patient in favour of the genotype-guided arm, which did not reach statistical significance given the high inter-individual variability of hematological costs and the limited sample size. When costs were stratified by clinical outcome, the experimental arm showed a narrower cost distribution than the control arm, without high-cost outliers. These observations are exploratory and hypothesis-generating, and they support further evaluation of pre-emptive *CYP2C19* genotyping as a potentially cost-effective strategy in adequately powered multicentre studies (Verbelen et al., 2017).

We acknowledge the limitations of this study. The sample size (n = 32) limited the statistical power for the primary endpoint (1−β = 0.597). As a consequence, the analysis is exploratory and hypothesis-generating, and any inferential interpretation must be made with caution. Additionally, the open-label design was a safety necessity to allow clinicians to act on genotype results. Beyond the overall sample size, several specific limitations deserve to be acknowledged. First, although the present analysis considered body weight and allometric scaling in the discussion of pediatric voriconazole pharmacokinetics, it did not perform any formal stratification by paediatric age subgroups, nor a detailed evaluation of age-related developmental pharmacokinetics (maturational changes in hepatic ontogeny and in CYP-mediated metabolism). With sixteen patients per arm, any such stratification would have produced subgroups too small to support a methodologically meaningful evaluation, and we therefore explicitly acknowledge this as an additional limitation. Second, the study was not powered to detect differences in economic outcomes; the cost-consequence findings should accordingly be interpreted with the same caution as the other exploratory results. Third, in pediatric hematology and HSCT populations, systemic inflammation may induce phenoconversion of CYP-mediated drug metabolism and contribute to the variability of voriconazole exposure independently of the *CYP2C19* genotype. Inflammatory markers were not collected in this trial, which precludes an evaluation of their potential contribution to the observed variability and should be explicitly recognised as a further limitation, to be addressed in future studies. However, the randomized controlled nature of the trial, the rigorous centralization of bioanalysis, and the strict adherence to established guidelines (CPIC, ECIL-6) provide a high level of evidence often missing in pediatric pharmacogenetics.

Additional sources of variability in voriconazole exposure that were not specifically captured in this analysis include drug–drug interactions with immunosuppressants, chemotherapeutic agents, proton pump inhibitors and other CYP modulators frequently used in this population, as well as variations related to disease severity. The route of administration (intravenous versus oral) and the clinical indication (prophylaxis versus targeted therapy) were not pre-specified as data to be collected, in line with the pragmatic design of the trial and with the bioequivalence between oral and intravenous voriconazole at recommended pediatric maintenance doses, as stated in the corresponding summary of product characteristics. Because the primary endpoint is a pharmacokinetic variable common to both indications and routes, our analysis shows that, under these pragmatic conditions, a pre-emptive CYP2C19 genotype-guided strategy is associated with a lower proportion of early subtherapeutic exposure and with no signal of increased direct medical costs compared with standard of care.

The VORIGENIPHARM trial presented here should be interpreted as a small randomized pilot study, whose recruitment could not reach the planned sample size, as the COVID-19 pandemic delayed its initiation within a fixed-duration public grant. In this exploratory cohort, pre-emptive *CYP2C19* genotype-guided dosing was associated with a lower proportion of early subtherapeutic voriconazole exposure compared with standard weight-based dosing, with no signal of increased toxicity. The observed economic signal pointed in the same direction but did not reach statistical significance. These results do not provide definitive evidence of clinical efficacy or cost-effectiveness; they are exploratory and hypothesis-generating, and they support the need for adequately powered multicentre studies to confirm these preliminary observations.

## Data Availability

The datasets generated and analysed during the current study are deposited in the Zenodo repository (DOI: 10.5281/zenodo.21306941; https://zenodo.org/records/21306941). Owing to the small size of the cohort and the sensitive nature of paediatric clinical and genetic data, access to the individual-level dataset is subject to controlled access and may require a data access agreement, in accordance with the study’s ethical approval (Clinical Research Ethics Committee of La Paz University Hospital, internal code 5316) and applicable data protection legislation. Requests to access the datasets should be directed to the corresponding authors.
